# Prevalence and antimicrobial susceptibility profiles of *Staphylococcus aureus* nasal carriage among pre-clinical and clinical medical students in a Tanzanian University

**DOI:** 10.1186/s13104-016-1858-0

**Published:** 2016-01-27

**Authors:** Benard Okamo, Nyambura Moremi, Jeremiah Seni, Mariam M. Mirambo, Benson R. Kidenya, Stephen E. Mshana

**Affiliations:** Department of Microbiology and Immunology, Catholic University of Health and Allied Sciences, Bugando, P.O Box 1464, Mwanza, Tanzania; Department of Biochemistry and Molecular Biology, Catholic University of Health and Allied Sciences, Bugando, Mwanza, Tanzania

**Keywords:** Antimicrobial susceptibility, *Staphylococcus aureus* nasal carriage, Medical students, Tanzania

## Abstract

**Background:**

Methicillin resistant *Staphylococcus aureus* (MRSA) nasal carriage is a potential niche for spread and a risk factor for subsequent infections. Despite the fact that medical students are exposed to patients in the hospital during their training, information on *S. aureus* and MRSA nasal carriage among medical students in Tanzania remains to be dearth so as to guide appropriate infection control and preventive measures.

**Methods:**

A cross-sectional study involving 314 medical students, pre-clinical (n = 166) and clinical (n = 148), at Catholic University of Health and Allied Sciences (CUHAS) was conducted from February to June 2013. Nasal swabs from eligible students were taken and processed using standard operating procedures so as to identify *S. aureus*, MRSA and their respective antimicrobial susceptibility patterns.

**Results:**

The median age (interquartile range) of the study participants was 24 (22–27) years with approximately 69.4 % being males. *S. aureus* accounted for 21.0 % (66/314) of which 1.5 % (1/66) was MRSA; giving an overall MRSA nasal carriage prevalence of 0.3 % (1/314). *Staphylococcus aureus* carriage among pre-clinical and clinical students were 19.9 % (33/166) and 22.3 % (33/148) respectively. MRSA carriage was found in one preclinical student with history of working in hospital for years prior to join CUHAS. *Staphylococcus aureus* carriage was significantly more in older median age group among clinical students compared to preclinical students (p < 0.001). Majority of the isolates were resistant to Ampicillin (87.9 %, 58/66) while all were sensitive to Ciprofloxacin and Vancomycin.

**Conclusion:**

There is high prevalence of *S. aureus* carriage among medical students at CUHAS. Fortunately, MRSA was found in only one student. In the light of these findings, focused MRSA surveillance to other potential sources like health care workers, patients and environment should be carried out in this setting.

**Electronic supplementary material:**

The online version of this article (doi:10.1186/s13104-016-1858-0) contains supplementary material, which is available to authorized users.

## Background

*Staphylococcus aureus* is considered as one of the most frequently occurring bacterial pathogens in the community and hospital settings and is associated with various forms of infections, ranging from mild skin infection to fatal invasive infections like fulminant septicemia [1–4]. Methicillin-resistant *Staphylococcus aureus* (MRSA) is a strain of *S. aureus* which is resistant to beta-lactam antibiotics resulting into treatment challenges, increased morbidity and mortality [4–7].

Up to 20 % of healthy individuals may be persistent carriers of *S. aureus* for periods ranging from a few weeks to many years and mostly in the anterior nares [8]. It is well known that *S. aureus* and MRSA nasal carriage is a potential niche for their continuous spread in the community and hospital environment as well as a risk factor for subsequent infections among individual carrying these strains [4, 8, 9]. Various studies have shown that individuals exposed to the hospital environment for prolonged period of time like healthcare workers and medical students have increased chances of carrying these strains and can be potential source of nosocomial infections [10–13]. This is contrary to people in the general community where there is no or very low MRSA carriage rate [14, 15]. Apart from exposure to hospital environment and MRSA nasal carriage, other risk factors for Staphylococcal infections reported in various studies are diabetes mellitus, extremes of ages (under-five children and individuals more than 65 years), indwelling devices, intravenous drug users and immunocompromized state [4, 8, 16, 17].

The antimicrobial resistance trend at Bugando Medical Centre (BMC) is growing and mostly being attributable to multidrug resistance bacteria; with reports revealing the proportion of MRSA among *S. aureus* isolates raising from 16.3 % in 2009 to 44.4 % in 2014 [5, 18, 19]. Reports in this hospital are showing association of MRSA with mortality among neonates and moreover, there is clonal spread of the predominant genotypes namely ST88 and new ST1797 among patients with skin and soft tissue infections (SSTIs) in different wards [5, 20]. This in turn necessitated a need to delineate potential sources of MRSA so as to control their spread. Despite the fact that medical students at Catholic University of Health and Allied Sciences (CUHAS) are exposed to patients as part and parcel of their training at BMC, information on *S. aureus* and MRSA nasal carriage among them remains to be dearth so as to guide appropriate infection control and preventive measures. We hypothesized that clinical students would be more colonized by MRSA due to their frequent visits to the wards and clinics than pre-clinical students. The specific objectives were to determine the prevalence of *S. aureus* and MRSA nasal carriage among medical students at CUHAS, the antimicrobial susceptibility profiles of *S. aureus* isolates and to ascertain the association of *S. aureus* nasal carriage with demographic and clinical characteristics.

## Methods

### Study design and sampling procedures

This was a cross-sectional study conducted between February and June 2013 at CUHAS; which is a higher learning institution situated within the BMC (a referral, consultant and teaching hospital) in the northwestern Tanzania. Using Kish-Leslie formula and a prevalence of 50 % (as there was no similar study previously conducted in Tanzania), a minimum sample size of 385 medical students was targeted. However, we were able to enroll randomly 314 medical students taking the courses of Medicine (MD) and Bachelor of Medical Laboratory Sciences (BMLS). These were divided into two groups namely pre-clinical (n = 166, out of 169 eligible students) and clinical (n = 148, out of 225 eligible students). Pre-clinical medical students included second year MD and BMLS while clinical medical students included fourth and fifth year MD as well as third year BMLS.

### Sample collection and laboratory procedures

Demographic and clinical information from consenting students were collected using pretested questionnaires (Additional file 1). A sterile cotton swab was moistened by inserting into sterile 0.85 % saline solution in universal bottle and then extra liquid expressed out by compressing it against the sides of the universal bottle. The moistened swab was then inserted into anterior nares, one at a time with the same swab, and rotated gently against the inner surface. The swab was withdrawn and immediately streaked on Sheep Blood Agar (HIMEDIA, Mumbai, India). The plates were then incubated at 35–37 °C for 24 h. Initial *S. aureus* screening was based on the presence of golden yellowish or creamy white colonies with or without beta hemolysis. These were further identified by Gram staining reaction and catalase test, followed by phenotypic confirmation using slide and tube coagulase as well as DNAse tests [21].

Antimicrobial testing on Muller Hinton Agar (HIMEDIA, Mumbai, India) was performed using the disc diffusion method as previously described by Clinical Laboratory Standard Institute [22]. The Standard antimicrobial disks (OXOID, UK) used were ampicillin (10 units), cefoxitin (30 μg), ciprofloxacin (5 μg), tetracycline (30 μg), erythromycin (15 μg) and vancomycin (30 μg). Moreover, isolates with zone of inhibition of ≤21 mm to cefoxitin (30 μg) were considered to be MRSA phenotypically [22].

### Quality control

*Staphylococcus aureus* ATCC 25923 and *S. epidermidis* ATCC 12228 were used as positive and negative controls for phenotypic identification of *S. aureus*.

### Data management and analysis

Every participant had a unique identification number. The demographic, clinical data and laboratory results in the questionnaires and laboratory log book were entered into Microsoft excel for consistent checks and data cleaning. These in turn were exported into STATA software version 11.0 for analysis according to the objectives of the study. Continuous variables were described as median (interquartile range). Categorical variables were described as proportions and were analyzed to compare the significance of difference in distribution by using Chi square test or Fischer’s exact test where appropriate. The difference in distribution was considered significant if p value was less than 0.05.

### Study clearance and ethical considerations

This study was conducted in accordance with the Declaration of Helsinki. Ethical clearance was sought and provided by Catholic University of Health and Allied Sciences/Bugando Medical centre Ethics and review committee. Written informed consent was sought voluntarily from each participant prior to collect information and nasal swab.

## Results

A total of 314 students were enrolled into the study. Of these, 218 (69.4 %) were males. The median age (IQR) was 24 (22–27) years, ranging from 19 to 43 years. Majority of participants were MD students (271, 86.3 %) while the rest were BMLS students. Irrespective of the courses, the pre-clinical students were 166 (52.9 %) whereas clinical students were 148 (47.1 %) (Table 1).

**Table 1:** Demographic and clinical characteristics of participants

Variable	Frequency	Percent (%)
Sex		
Male	218	69.4
Female	96	30.6
Course		
MD	271	86.3
BMLS	43	13.7
Status of students		
Clinical	148	47.1
Pre-clinical	166	52.9
Antibiotic use^a^		
Yes	36	11.5
No	278	88.5
Hospital admission^b^		
Yes	7	2.2
No	307	97.8
Exposure to SSTIs		
Yes	3	1
No	311	99

*MD* Doctor of medicine, *BMLS* Bachelor of Medical Laboratory Sciences, *SSTIs* skin and soft tissue infections

^a^In the past 2 weeks

^b^In the past 3 months

The overall carriage rate of *S. aureus* was 21.0 % (66/314). Of these, MSSA accounted for 65 (98.5 %) isolates whereas only 1 (1.5 %) isolate was found to be MRSA; giving the prevalence of MRSA nasal carriage among all students to be 0.3 % (1/314). Majority of the colonized students were pursuing MD course, 57 (86.4 %) with only 9 (13.6 %) pursuing BMLS course. There was a numerical similarity between colonized preclinical and clinical students with each comprising of 33 (50 %), giving the overall carriage rates among pre-clinical and clinical students to be 19.9 % (33/166) and 22.3 % (33/148) respectively. Most of the colonized students had no history of antibiotic use in the past 2 weeks (90.9 %), no history of previous hospital admission in the past 3 months (98.5 %) and with no exposure to SSTIs (100.0 %). Nevertheless, none of these variables had a statistical significant association in relation to *S. aureus* carriage (Table 2).

**Table 2:** Association of Students’ *S. aureus* carriage with demographic and clinical characteristics

Variable	*S. aureus* carriage		p value
	Yes (N = 66) n (%)	No (N = 248) n (%)	
Age			
Median (IQR)	24.5 (23–28)^a^	24 (22–26.5)^a^	0.1323
Gender			
Male	43 (65.2)	175 (70.6)	0.396
Female	23 (34.8)	73 (29.4)	
Course			
MD	57 (86.4)	214 (86.3)	0.988
BMLS	9 (13.6)	34 (13.7)	
Clinical			
Yes	33 (50.0)	115 (46.4)	0.600
No	33 (50.0)	133 (53.5)	
Antibiotic use^b^			
Yes	6 (9.1)	30 (12.1)	0.496
No	60 (90.9)	218 (87.9)	
Hospital admission^c^			
Yes	1 (1.5)	6 (2.4)	0.548
No	65 (98.5)	242 (97.6)	
Exposure to SSTIs			
Yes	0 (0.0)	3 (1.2)	0.491
No	66 (100)	245 (98.8)	

*MD* Doctor of medicine, *BMLS* Bachelor of Medical Laboratory Sciences, *SSTIs* skin and soft tissue infections

^a^Continuous variable

^b^In the past 2 weeks

^c^In the past 3 months

Of all the 66 *S. aureus* isolates, 31 (47 %) were from preclinical students while 35 (53 %) were from clinical students. Of all colonized preclinical students, none had history of antibiotic use in the past 2 weeks, history of hospital admission in the past 3 months and history of exposure to SSTIs. Only 1 (3 %) of the colonized clinical students had been admitted in hospital within 3 months and also had history of antibiotic use in the past 2 weeks prior to the commencement of the present study. *Staphylococcus aureus* carriage was significantly more in older median age group among clinical students compared to preclinical students (p < 0.001). There was no significant association between the carriage of *S. aureus* between clinical and pre-clinical students with all other predictor variables (Table 3).

**Table 3:** Distribution of *S. aureus* carriage between preclinical and clinical students

Variable	*S. aureus* carriage		p value
	Preclinical (N = 33) n (%)	Clinical (N = 33) n (%)	
Age			
Median (IQR)	23 (22–25)^a^	25 (24–28)^a^	<0.001
Gender			
Male	21 (63.6)	22 (66.7)	0.796
Female	12 (36.4)	11 (33.3)	
Course			
MD	29 (87.9)	28 (84.8)	0.500
BMLS	4 (12.1)	5 (15.2)	
Antibiotic use^b^			
Yes	5 (15.2)	1 (3.0)	0.098
No	28 (84.8)	32 (97.0)	
Hospital admission^c^			
Yes	0 (0.0)	1 (3.0)	0.500
No	33 (100.0)	32 (97.0)	
Exposure to SSTIs			
Yes	0 (0.0)	0 (0.0)	–
No	33 (100.0)	33 (100.0)	

*MD* Doctor of medicine, *BMLS* Bachelor of Medical Laboratory Sciences, *SSTIs* skin and soft tissue infections

^a^Continuous variable

^b^In the past 2 weeks

^c^In the past 3 months

**Fig. 1:**
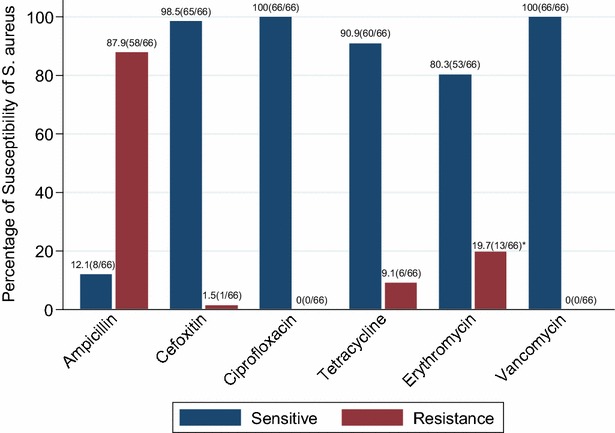
Antimicrobial susceptibility profiles of *S. aureus* isolates. Of the 13 resistance *S. aureus* isolates to erythromycin, five had intermediate resistance

More than three quarter of *S. aureus* isolates (87.9 %, 58/66) were resistant to Ampicillin whereas only one isolate (1.5 %) was resistant to Cefoxitin (i.e., MRSA isolate). All isolates were sensitive to Ciprofloxacin and Vancomycin (Fig. 1).

## Discussion

The prevalence of *S. aureus* and MRSA nasal carriage among medical students at CUHAS was 21.0 and 0.3 % respectively; with *S. aureus* carriage among preclinical and clinical students accounting for 19.9 and 22.3 % respectively. The finding on *S. aureus* nasal carriage is comparable with previous similar study from Taiwan (19.3 %) and China (23.1 %) [13, 23]. Even though clinical students in both studies were more colonized than pre-clinical students connoting the role of exposure in the former group, there was no statistical significant different between the two groups. The prevalence of MRSA from this study is relatively low compared to similar studies in Taiwan (2.2 %) and USA (2.0 %) but contrary to these studies, a study in Australia showed no MRSA carriage [23–25]. The variation could be attributable to different geographical locations, extent of risky environmental exposures as well as different infection control and prevention policies across countries as reported from other studies where the rates were as high as 6.2–9.7 % among health care workers as opposed to the general population [10, 11, 14].

The median age in the present study was significantly higher among clinical students carrying *S. aureus* than the pre-clinical students. Similarly, older age has been found in another study to be an independent predictor of *S. aureus* carriage [25]. These may be attributed to the increase in the likelihood of *S. aureus* exposure and subsequent acquisition in the hospital setting. We had hypothesized that clinical students would be more colonized by MRSA due to their frequent visits to the wards and clinics where MRSA prevails than pre-clinical students but contrary to this, the MRSA isolate was isolated from a preclinical student. The plausible explanation could be the fact that this student was working in the hospital for several years prior to join CUHAS, where he could have potentially acquired this strain. Despite the fact that the present study found MRSA carriage in only one student (0.3 %); the prevailing high rate of MRSA infections, morbidity and mortality associated with these strains among patients at BMC as reported from previous studies in Mwanza, Tanzania [5, 26] and in other countries [1, 6, 8, 16] calls for continuous MRSA surveillance so as to find out the contribution of health workers and environment as potential alternative sources.

There was no significant difference between the carriers and non-carriers of *S. aureus* in terms of different courses, sex, clinical exposure, having family or close relative with SSTIs, history of hospital admission and exposure to antibiotics. Though not statistically significant, this study and other similar studies found the preponderance of colonization by *S. aureus* among male students as compared to female students [15, 23–25]. Personal hygiene and genetic aspects may account on the gender differences in relation to *S. aureus* nasal carriage status. Moreover, a systematic review has clearly shown a plausible association between antibiotic exposure and MRSA carriage [27].

The antimicrobial resistance profiles shown in the present study is comparable to many other studies within and outside Tanzania reiterating the fact that pathogens are likely to develop resistance to most commonly used drugs (ampicillin and erythromycin) as opposed to less commonly used drugs (ciprofloxacin and vancomycin) [6, 17, 18, 25, 28]. Colonization with *S. aureus* strains with paucity or no resistance as shown in the present study is good because these normal flora can protect individuals against pathogenic bacteria [12, 14]. In the light of these, feasible measures at predetermined intervals should be focused on MRSA surveillance to other potential sources like health care workers, patients and environment [8, 14, 17]. This in turn will prevent subsequent invasive and deadly infections [5, 7, 9]. Finally, policies/guidelines on restriction of individuals’ colonized with MRSA and decolonization strategies should be established in this setting. This is especially important when colonized individual attend risky patients such as those with surgical site infections, neonates as well as patients admitted in intensive care unit.

## Limitation

The small sample size for comparison purposes between preclinical and clinical medical students may have affected the statistical power of comparison, despite this baseline information on *S. aureus* nasal carriage among medical students at CUHAS has been laid down for future assessment of change in trends.

## Conclusion

There is high prevalence of *S. aureus* carriage (21.0 %) among medical students at CUHAS. Fortunately, MRSA was found in only one preclinical student. In the light of these findings, focused MRSA surveillance to other potential sources like health care workers, patients and environment should be carried out in this setting so as to further identify the niches of these strains and confer specific infection control and prevention measures. A large sample size across all medical universities in Tanzania would be of interest to further delineate the trend of MRSA carriage throughout the country (Additional file 2).

## Additional files

10.1186/s13104-016-1858-0 Questionnaire_English version.
10.1186/s13104-016-1858-0 STROBE_checklist_cross-sectional.

## Abbreviations

BMC: Bugando Medical Centre
BMLS: Bachelor of Medical Laboratory Sciences
CUHAS: Catholic University of Health and Allied Sciences
MD: Doctor of Medicine
MRSA: methicillin resistant *Staphylococcus aureus*
SSTIs: skin and soft tissue infections
