# Supplementary material for: Prevalence and antimicrobial susceptibility profiles of Staphylococcus aureus nasal carriage among pre-clinical and clinical medical students in a Tanzanian University
Source: BMC Res Notes. 2016 Jan 27;9:47. doi: 10.1186/s13104-016-1858-0 (PMC4728816; doi:10.1186/s13104-016-1858-0)
Supplement: Supplementary file 1 — 10.1186/s13104-016-1858-0 Questionnaire_English version. [file 13104_2016_1858_MOESM1_ESM.doc]

Additional file I: **Questionnaire_English version**

**Section A: Socio-demographic and clinical characteristics**

1. Date………………………….
2. Study code…………………….
3. Sex i. Female ii. Male
4. Age ……………………(years)
5. Course……………………..
6. Year of study……………….
7. Any history of antibiotic(s) use in the past 2 weeks? i. Yes ii. No
8. If yes, what type of antibiotic(s)? …………………………………………….
9. Any history of hospital admission in the past 3 months? i. Yes ii. No
10. If yes, what was the duration? ………………………… ……………….(days)
11. Do you have relative/ friend/ roommate with Soft Tissue Infection (SSTI)? i. Yes ii. No

**Section B: Laboratory results**

1. Bacterial growth on sheep blood agar i. Yes ii. No
2. Hemolysis on sheep blood agar i. Yes ii. No
3. Color of colonies on sheep blood agar i. Golden yellow ii. Creamy iii. Others (specify…………………)
4. Catalase test i. Positive ii. Negative
5. Coagulase test i. Positive ii. Negative
6. DNase test i. Positive ii. Negative
7. *Staphylococcus aureus* confirmed phenotypically i. Yes ii. No
8. Antimicrobial susceptibility testing:

| Antimicrobial agent | Diameter (cm) | Sensitive/Intermediate/Resistance |
| --- | --- | --- |
| Ampicillin (10 μg) |  |  |
| Cefoxitin (30 μg) |  |  |
| Ciprofloxacin (5 μg) |  |  |
| Tetracycline (30 μg) |  |  |
| Erythromycin (15 μg) |  |  |
| Vancomycin (30 μg) |  |  |

1. Methicillin resistant *Staphylococcus aureus* (MRSA) isolate i. Yes ii. No
